# Study of 3C-SiC Power MOSFETs

**DOI:** 10.3390/mi16121406

**Published:** 2025-12-14

**Authors:** Hamid Fardi

**Affiliations:** Department of Electrical Engineering, University of Colorado Denver-Anschutz Denver, Aurora, CO 80204, USA; hamid.fardi@ucdenver.edu

**Keywords:** silicon carbide, power MOSFETs, breakdown field, avalanche ionization

## Abstract

This work presents the simulation and design of 3C-SiC power MOSFETs, focusing on critical parameters including avalanche impact ionization, breakdown voltage, bulk and channel mobilities, and the trade-off between on-resistance and breakdown voltage. The device design is carried out by evaluating the blocking voltage of scaled structures as a function of the blocking layer’s doping concentration. To mitigate edge-effect breakdown at the p-well/n-drift interface, a step-profile doping strategy is employed. Multiple transistor layouts with varying pitches are developed using a commercially available device simulator. Results are benchmarked against a one-dimensional analytical model, validating the on-state resistance, current–voltage behavior, and overall accuracy of the simulation approach. For the selected material properties, simulations predict that a 600 V 3C-SiC MOSFET achieves an on-state resistance of 0.8 mΩ·cm^2^, corresponding to a 7 μm drift layer with a doping concentration of 1 × 10^16^ cm^−3^.

## 1. Introduction

Recent progress in the epitaxial growth of high-quality 3C-SiC [1,2,3,4] has revived strong interest in cubic silicon carbide as a promising semiconductor for power electronics. Compared to conventional silicon, all SiC polytypes demonstrate superior intrinsic material properties, including lower intrinsic carrier concentration, higher critical breakdown field, greater thermal conductivity, and higher electron saturation drift velocity. These attributes enable the design of power devices with thinner drift regions for a given voltage rating, which in turn lowers the specific on-resistance. A reduced on-resistance translates directly into lower conduction losses and improved energy efficiency, making SiC a compelling alternative to silicon in high-performance power switching applications.

Within the family of SiC polytypes, 3C-SiC offers a particularly attractive advantage: a channel mobility nearly five times greater than that of 4H-SiC, reaching values of approximately 380 cm^2^/V·s [5]. Higher channel mobility directly reduces channel resistance, thereby further lowering the total on-resistance—provided that the material also sustains a sufficiently high breakdown field. This combination positions 3C-SiC as a highly promising candidate for MOSFET design, especially in applications demanding both low on-state resistance and reduced switching losses.

Significant achievements have already been demonstrated with 4H-SiC MOSFETs, where continuous improvements in material quality and device processing have led to steady gains in blocking capability. State-of-the-art 4H-SiC devices report a specific on-resistance as low as 2.7 mΩ·cm^2^ for a blocking voltage of 1.2 kV [6], while other studies indicate values ranging from 5 to 90 mΩ·cm^2^. By contrast, the development of 3C-SiC MOSFETs remains less advanced. Their performance is strongly influenced by the chosen material parameters, and progress is limited by the scarcity of reliable experimental data.

To overcome these challenges, this paper adopts a hybrid approach: carefully selected material properties are drawn from literature and supplemented with simulation-based estimates when experimental data are lacking. These values are then incorporated into a comprehensive device model, enabling systematic simulation of 3C-SiC MOSFETs and extraction of their key performance metrics. Through this methodology, we provide insights into the viability of 3C-SiC for next-generation power electronics and evaluate its potential advantages over more established SiC polytypes. This approach was used in an earlier study to realize the ionization rate electric field relationship in [7] and bulk mobility doping density in [8] for 3C-SiC.

## 2. Device Structure and Physical Parameters

**Figure 1:**
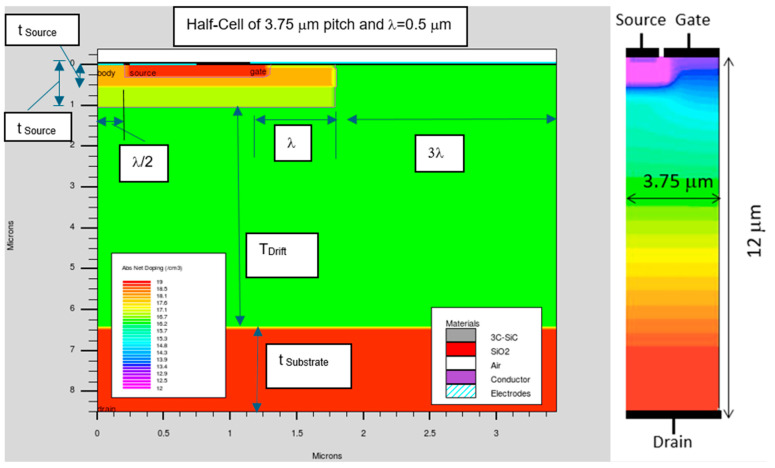
Half-cell of the vertical power MOSFET structure. All dimensions shown are in micrometers; other values are specified in Table 1.

In this study, the power device under consideration is a vertical MOSFET designed with a lateral electron conduction channel. The representative half-cell geometry is depicted in Figure 1, which highlights the critical features of the simulated structure. To account for realistic fabrication constraints, the gate-to-source overlap is taken to be 0.1 μm, consistent with values achievable through a self-aligned source formation process.

The active cell layout is composed of two primary regions: a MOSFET channel region with a length of 1.75 μm and a JFET constriction region whose length is systematically varied to 1.5, 2.0, and 2.5 μm in order to evaluate its effect on device performance. For consistency, all devices are normalized to a total active area of 1 cm^2^, enabling direct comparison of their electrical characteristics.

The inclusion of the JFET region results in three distinct device geometries characterized by pitch sizes of 3.25, 3.75, and 4.25 μm. These correspond to calculated cell widths of 3077 cm, 2667 cm, and 2353 cm, respectively. Despite these structural variations, the thickness of the gate oxide is held constant at 50 nm for all cases, ensuring that changes in device performance can be attributed primarily to variations in the cell geometry and JFET length.

A complete summary of the baseline material parameters and device design specifications employed in the simulations is provided in Table 1. These parameters form the foundation for the numerical analysis and allow the investigation of scaling trends and structure–performance trade-offs across the simulated 3C-SiC MOSFET devices.

At the time of this investigation, experimental data relevant to high-power, high-blocking device applications were available, including detailed measurements of device breakdown voltage and specific on-resistance. These data provided a useful basis for validating and comparing the simulation results presented in this study.

### 2.1. Influence of Doping on Low-Field Mobility

The performance of a power MOSFET is primarily governed by three material-dependent quantities: (i) the bulk electron mobility in the drift region, (ii) the critical breakdown field, and (iii) the channel mobility at the SiC/oxide interface. Among these, the doping dependence of bulk electron mobility is particularly important, as it directly impacts drift-layer resistance.

In this study, bulk mobility is modeled using the conventional empirical relation given in [9], with parameter values summarized in Table 2.

(1) $$\mu_{0}=\mu_{\min}+\frac{\mu_{\max}-\mu_{\min}}{1+{\left(\frac{N}{N_{r}}\right)}^{\alpha }}$$

**Figure 2:**
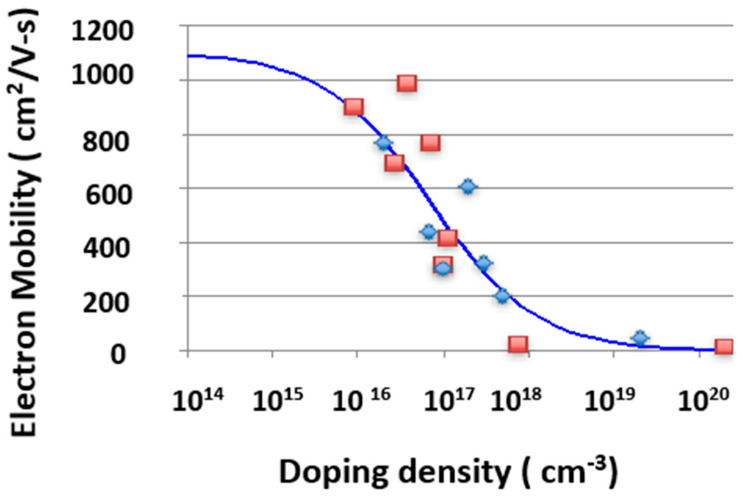
3C-SiC bulk electron mobility versus doping density fitted to experimental data taken from references [12,13,14,15].

For reference, the table also includes the mobility fitting constants for 4H-SiC, enabling side-by-side comparison. The fitting parameters for 4H-SiC are extracted from experimental measurements [10,11], while those for 3C-SiC are taken from several independent studies [12,13,14,15]. As shown in Figure 2, the resulting fit aligns closely with published experimental results. The slightly higher mobility observed for 3C-SiC is consistent with its marginally lower conductivity effective mass, as discussed in [16,17,18,19,20].

The mobility data for 3C-SiC are derived from two sources: bulk crystals [12] and high-quality epitaxial layers grown on silicon substrates [13,14,15]. To reduce scatter, preference is given to datasets obtained from lightly doped material, as indicated by the additional impurity concentration required to achieve p–n conversion. This approach ensures that the modeled mobility reflects intrinsic material behavior rather than impurity-limited conduction.

Experimentally reported channel mobilities for n-channel 3C-SiC MOSFETs vary widely, from as low as ~100 cm^2^/V·s [20] to as high as ~370 cm^2^/V·s [5] in devices fabricated on silicon-based 3C-SiC substrates. For high-voltage lateral MOSFETs, however, channel design requires moderate p-type doping to maintain threshold stability as the channel length is reduced. Taking this into account, a representative mobility value of 200 cm^2^/V·s is selected for the present study [5,21]. This choice balances experimental extremes and avoids dependence on peak values obtained under special conditions (e.g., very low doping, novel oxide stacks, or vertical channel geometries) that may compromise long-term device stability.

### 2.2. Doping Dependence of Breakdown Field and Blocking Voltage

The breakdown characteristics of 3C-SiC devices are strongly influenced by the doping concentration and thickness of the drift layer. In this work, breakdown voltage values are determined using published impact ionization coefficients for electrons and holes [22,23], combined with Atlas device simulations [24].

ATLAS automatically adjusts the step size in voltage or during bias sweep simulations to ensure convergence and accuracy (it is not the focus of this study), which is explained in detail in the user’s manual, ref. [24]. These simulations enable the extraction of the effective breakdown field and support the development of an empirical model suitable for 3C-SiC MOSFET design. The simulated breakdown behavior is compared against available experimental measurements for validation [7].

Ionization rates for electrons ($\alpha_{n}$) and holes ($\alpha_{p}$) are taken from Monte Carlo studies [25,26] and fitted to the standard exponential field-dependent expression [27]:

(2) $$\alpha_{n,p}=Ae^{-\frac{B}{E}}$$

where *A* and *B* are fitting parameters and *E* is the local electric field. Impact ionization coefficients often follow the exponential behavior of Equation (2). Real materials frequently show *two* regimes: a low-to-moderate field regime where carriers gain energy more slowly (stronger effective barrier, more phonon scattering) and a high-field regime where carrier heating and nonlocal acceleration make ionization much more probable. Splitting at **Ec** models the physical transition between those regimes. In addition, holes typically have larger effective mass, different scattering rates, and band-structure-dependent thresholds, so their ionization rate slope can change more markedly with field than for electrons.

Empirical ionization data often show a kink or slope change at a particular field for holes, which justifies separate parameter sets A and B below and above Ec. At high fields, the local-field approximation becomes less accurate since carriers travel significant distances between collisions, and using different high-field parameters compensates for nonlocal effects if a full nonlocal model (full MC) is not used.

The hole ionization coefficient is parameterized using two exponential segments separated at Ec = 1.75 MV/cm. A single exponential often cannot fit experimental $\alpha_{p}$(E) across the whole field range. Piecewise exponentials allow a close fit to measured low- and high-field behavior, improving prediction of breakdown voltage. This segmentation models the transition from the low-field, phonon-limited ionization regime to a high-field, carrier-heating dominated regime where the slope of $\alpha_{p}$(E) changes. The piecewise form provides an accurate empirical fit to measured ionization data and avoids unphysical extrapolation at extreme fields. A sharp, unbounded exponential can cause numerical stiffness and convergence problems in ATLAS near breakdown. By capping slope changes and smoothing the segment junction, gradient changes are controlled. The continuity at **Ec** is enforced as piecewise fits which results in important physics without heavy computational cost and ensuring numerical stability in ATLAS [24].

**Figure 3:**
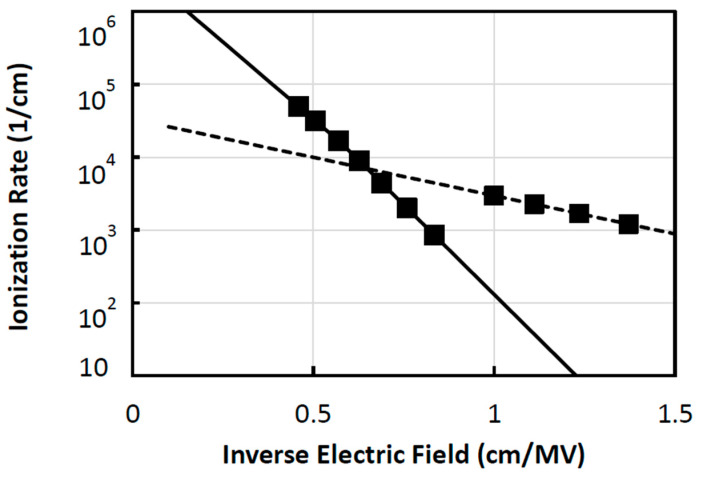
3C-SiC electron (dashed line) and hole (solid line) ionization rates versus inverse electric field; Monte Carlo results are shown in the square markers (for both electrons and holes) [15,16].

The extracted constants are summarized in Table 3, with electron and hole ionization parameters exhibiting slight deviations above and below 1.75 MV/cm. The resulting field-dependent ionization curves are shown in Figure 3.

**Figure 4:**
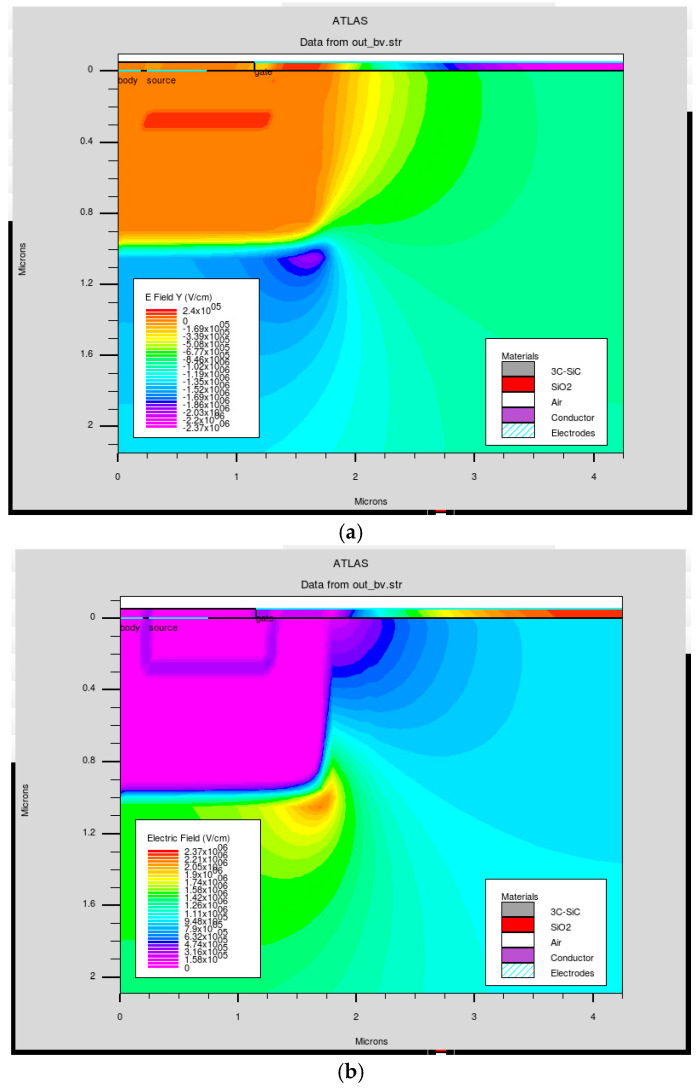
MOSFET electric field distribution for device No. 3, which has a pitch size of 4.25 μm. (**a**) Without a step-doping profile, a hot spot shows early, unwanted breakdown. (**b**) MOSFET with a step-doping profile does not show early breakdown voltage.

To suppress localized breakdown effects at the p-well/n-drift interface, a step-doping profile is implemented. As shown in Figure 4, this approach effectively eliminates hot-spot breakdown for device No. 3, which has a pitch size of 4.25 μm. The step profile begins with a high doping concentration of 1 × 10^18^ cm^−3^ over a 0.5 μm depth, followed by a reduced concentration of 1 × 10^17^ cm^−3^ over the next 0.5 μm. Additionally, a shallow 0.1 μm layer with 1 × 10^17^ cm^−3^ doping is introduced beneath the gate oxide to provide threshold voltage control.

All simulated devices assume a 2 μm-thick substrate with an n-type doping of 1 × 10^19^ cm^−3^. Under these conditions, substrate resistance is negligible. In practical devices, however, this resistance can dominate at low blocking voltages, requiring substrate thinning to mitigate its impact. Advances in SiC device fabrication have also significantly reduced contact resistance to the source and drain regions, allowing it to be neglected in power MOSFET analysis [28,29].

**Figure 5:**
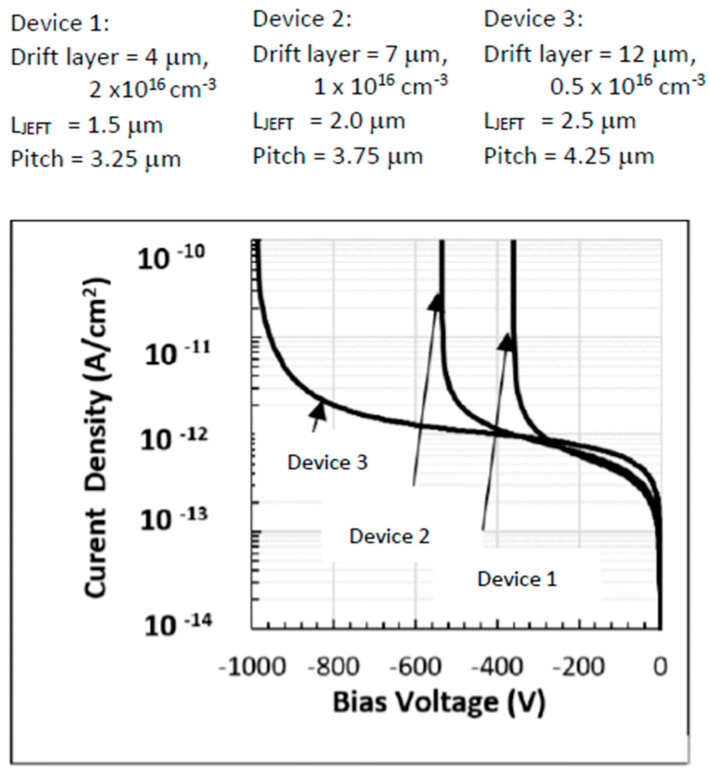
Two-dimensional simulation of breakdown voltages for devices No. 1–3 with different pitch sizes. Results are summarized in Table 4.

Two-dimensional simulations are employed to establish the optimal drift-layer doping concentration and thickness required to sustain a given blocking voltage. Figure 5 illustrates the blocking voltage as a function of these parameters for three different device geometries. A detailed comparison of the simulated breakdown voltages, drift-layer thicknesses, and corresponding doping levels is provided in Table 4, alongside values predicted by a one-dimensional depletion approximation. Deviations between the 1D and 2D models are attributed to lateral field crowding effects, which become more pronounced in devices with smaller pitch sizes.

The device behavior is modeled using a 1D variable depletion-layer model [9], which incorporates channel length modulation. In this framework, the lateral n-channel MOSFET is analyzed separately from the vertical JFET and drift region contributions, with the body and source terminals tied to eliminate the body effect. Because the 1D model does not capture the 2D field distribution, its predictions are benchmarked against full 2D Atlas simulations to ensure accuracy.

## 3. Simulation Results

### 3.1. Breakdown Field and Blocking Voltage

**Figure 6:**
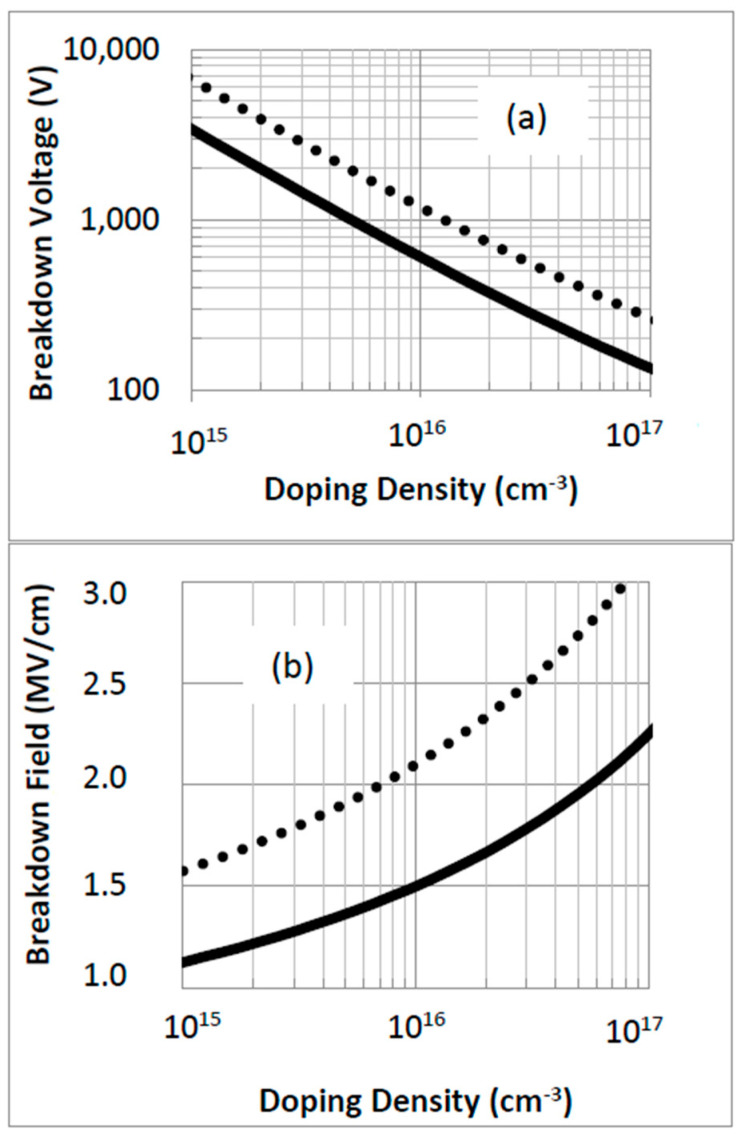
Maximum breakdown voltage (**a**) and breakdown field (**b**) of n-type 3C-SiC (solid line) and 4H-SiC (dashed line) versus doping density.

The simulated breakdown characteristics of 3C-SiC MOSFETs are summarized in Figure 6, where both the critical breakdown field (E_br_) and the corresponding breakdown voltage (V**_br_**) are plotted as a function of drift-layer doping. These quantities are connected through standard full-depletion approximations traditionally applied to silicon devices [30]:

(3) $$E_{br}=\sqrt{\frac{2qN_{d}V_{br}}{\varepsilon_{s}}},$$

And the equation fitted to the experimental data

(4) $$E_{br}=\frac{\varepsilon_{s}}{1-\frac{1}{3}\log\frac{N_{d}}{10^{16}{\mathrm{cm}}^{-3}}},$$

where Nd is the doping concentration of the drift layer, q is the electronic charge, and e_s_ is the dielectric constant of 3C-SiC. The relevant physical parameters used in the calculations are listed in Table 1, which also includes a direct comparison with values reported for 4H-SiC [7,8].

The simulated breakdown fields for 3C-SiC fall within the experimentally observed range of 1–2.5 MV/cm [11,12]. From the results, a MOSFET designed to block 600 V requires a drift-layer doping concentration of approximately 1.3 × 10^16^ cm^−3^. Although 3C-SiC offers marginally higher electron mobility than 4H-SiC, the latter retains a distinct advantage in minimizing drift resistance due to its higher critical breakdown field. This trade-off places practical limits on how low the on-resistance can be pushed in cubic SiC compared with its hexagonal counterpart.

### 3.2. DC Simulation of Current–Voltage Characteristics

**Figure 7:**
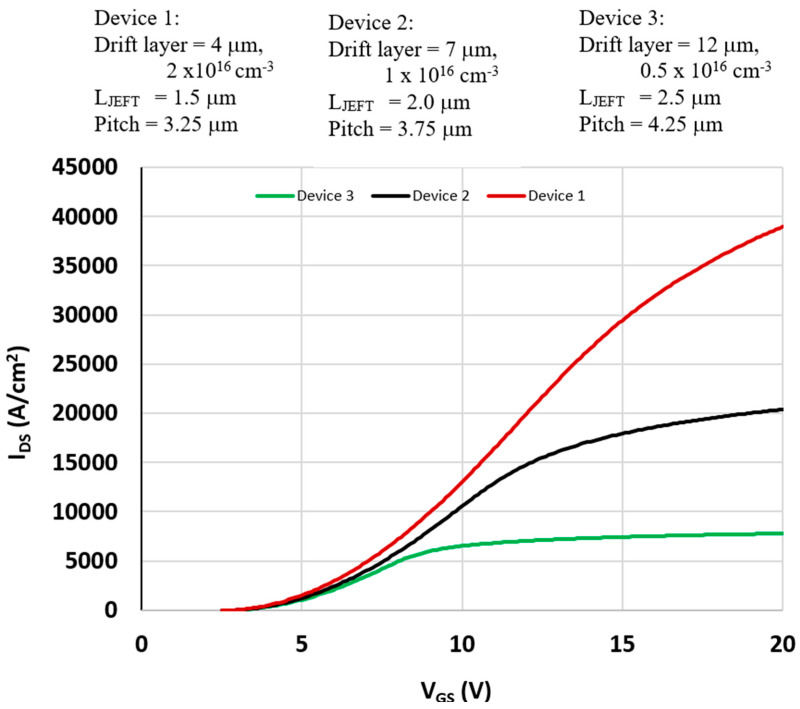
IDS-VGS (VDS = VGS) characteristics showing threshold voltage for devices No. 1–3 with different pitches.

A series of 2D device simulations were conducted to study the drain current response as a function of gate and drain bias for devices No. 1–3, with different cell pitches of 3.25, 3.75, and 4.25 μm. The IDS–VGS characteristics are shown in Figure 7, where the drain and gate voltages are swept together to examine conduction behavior across the three device geometries.

The objective of this work is not to match or reproduce the current–voltage characteristics of a specific fabricated 3C-SiC MOSFET, but rather to obtain an overall assessment of device performance—particularly breakdown voltage and specific on-resistance—using analytical models and 2-D numerical simulations. Direct calibration to experimental IDS–VGS curves is not performed because reliable, process-matched electrical data for 3C-SiC MOSFETs are not available for the epitaxial conditions considered here. In addition, the electrical characteristics of 3C-SiC devices depend strongly on factors such as substrate quality, defect density, doping-profile variations, low-field mobility, and interface trap distributions, all of which exhibit significant variability in the reported literature. Without access to the corresponding process details, fitting simulation models to any single dataset would introduce artificial tuning and reduce the generality of the results. Instead, well-established physical parameters and ionization-rate models from the literature are used to capture the underlying device physics and to evaluate performance trends. The conclusions presented are therefore based on the physical behavior and relative trends of 3C-SiC MOSFET structures rather than the calibration of a specific device.

Because each MOSFET is normalized to a unit die area of 1 cm^2^, the extracted current densities appear large. Consequently, the results should be interpreted on a per-area basis rather than per-device width. Device widths corresponding to each pitch are listed in Table 1. The low-bias slope of the output curves reflects the combined contributions of MOSFET channel resistance and JFET constriction resistance. Differences in transconductance among the three devices primarily arise from the fact that higher breakdown voltage designs require lighter doping in the drift region, which raises the drift resistance.

The transfer characteristics suggest that, for most bias conditions, the MOSFETs operate in the triode (linear) region rather than in saturation. This is particularly true at higher gate voltages, where a significant portion of the applied drain–source voltage is dropped across the JFET resistance.

The simulated threshold voltage (**V_t_**) extracted from 2D calculations is ~2.8 V for all device variants. A first-order 1D long-channel model using:

(5) $$V_{t}=V_{FB}+2\phi_{F}+\frac{\sqrt{2\varepsilon_{s}qN_{a}(2\phi_{F}+V_{SB})}}{C_{ox}}$$

predicts a threshold of ~3.2 V for device No. 3 with a 4.25 μm pitch. The slightly lower threshold obtained from 2D simulations is attributed to incomplete ionization of dopants, which lowers the effective charge density and built-in potential.

Comparisons with analytical 1D calculations (adjusted to include drift and JFET resistances) show good agreement, validating the simulation framework. At high drain bias, however, short-channel effects and velocity saturation limit current growth, deviating from the quadratic dependence predicted by long-channel models.

(6a) $$I_{DS}=\mu_{n}C_{ox}(\frac{W}{L})[(V_{GS}-V_{t})V_{DS}-\frac{1}{2}V_{DS}^{2}],V_{DS}\leq V_{GS}-V_{t}$$

(6b) $$I_{DS}=\mu_{n}C_{ox}\left(\frac{W}{L}\right)\left[\frac{(V_{GS}-V_{t}{)}^{2}}{2}\right],V_{DS}\geq V_{GS}-V_{t}.$$

The channel velocity is modeled by:

(7) $$\mu_{n}(E)=\mu_{0}{\left[\frac{1}{1+{\left(\frac{\mu_{0}E}{v_{s}}\right)}^{\beta }}\right]}^{\frac{1}{\beta }},$$

where a saturation velocity of Vs = 2 × 10 ^7^ cm/s and β = 1 are used [31]. In Equation (7) μ_0_ is the low-field mobility calculated from Equation (1) [31,32]. These effects reduce the effective channel mobility as vertical fields increase, consistent with experimental observations.

**Figure 8:**
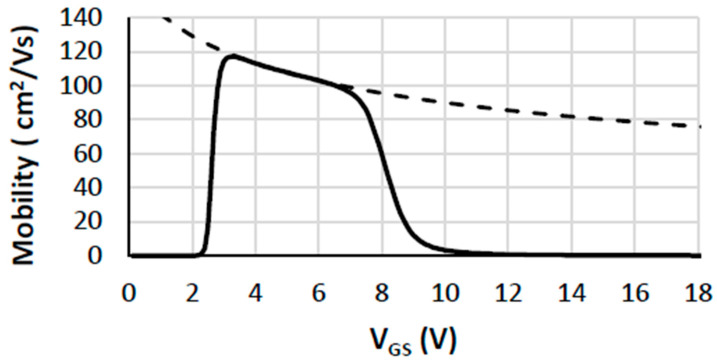
Mobility values as a function of gate voltage, extracted from 2D simulations. The dashed line shows the 1D analytical calculation for device No. 1 with a pitch size of 3.25 mm.

From the 2D results, device No. 1 (pitch = 3.25 μm) shows partial saturation behavior at gate biases between 5 and 10 V and a drain voltage of 20 V, allowing an effective channel mobility of ~115 cm^2^/V·s to be extracted (Figure 8). This is lower than the nominal 200 cm^2^/V·s assumed in the simulation model due to vertical field effects and carrier confinement (often described as carrier penetration or quantum confinement near the interface). Analytical 1D estimates yield an even lower effective mobility of ~71 cm^2^/V·s, reflecting model simplifications and incomplete ionization effects. Experimental values reported in the literature span 100–370 cm^2^/V·s [5,20,21,33,34,35,36,37], depending on device structure and doping profiles, highlighting the challenges in direct comparison.

### 3.3. Specific On-Resistance vs. Blocking Voltage

**Figure 9:**
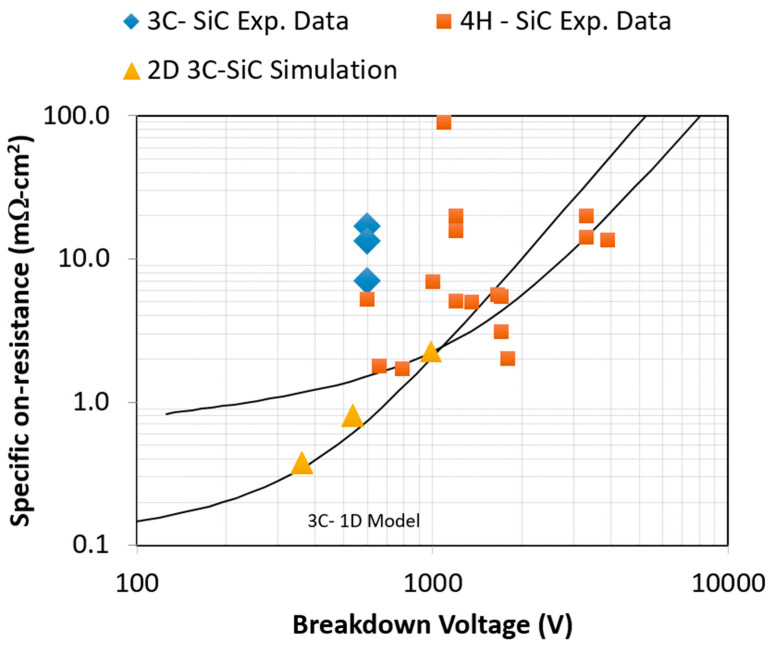
Two-dimensional simulated specific on-resistance versus breakdown voltage for devices No. 1–3 with pitch sizes of 3.25, 3.75, and 4.25 µm, compared with experimental data. Published 3C-SiC and 4H-SiC experimental data are included for reference. The 2D-simulated on-resistance values are summarized in Table 4, while Table 5 lists both the experimental data and the simulation results. The analytical 1D model (solid line) is also shown.

The output current-voltage characteristics are further analyzed to determine the specific on-state resistance of the simulated devices. By combining doping-dependent mobility with breakdown constraints, both 1D analytical models and 2D simulations are used to estimate resistance–voltage trade-offs. The results are summarized in Table 4 and plotted in Figure 9, which compares cubic and hexagonal SiC devices.

Figure 9 highlights the core focus of this work—the specific on-resistance of 2D-simulated 3C-SiC MOSFETs versus the breakdown voltage—and compares these results directly with published experimental data for vertical 3C-SiC MOSFETs. The experimental data and corresponding 2D simulation results, summarized in Table 5, provide the primary basis for evaluating the accuracy and relevance of the simulations.

The 2D simulations for 3C-SiC follow the general trend predicted by the 1D analytical model, while the available experimental data deviate from the simulated values, reflecting the technology’s relatively early stage of development. These discrepancies arise from uncertainties in doping profiles, epitaxial and interface quality, electron mobility, and trapping or surface-charge effects—factors that currently limit direct quantitative calibration between simulation and experiment for 3C-SiC.

Results for 4H-SiC are included for context; most reported values cluster around or above the 1D prediction and serve primarily as a mature-technology reference. The central insight of this comparison is the behavior of 3C-SiC: the 2D simulations capture key physical mechanisms and identify performance-limiting factors that align qualitatively with the emerging experimental landscape. Maintaining low channel doping to preserve mobility and current capability—while ensuring sufficient bulk doping to avoid punch-through—and gradually tapering the bulk doping toward the drift region remain essential design strategies for improving 3C-SiC device robustness and narrowing the gap between simulated and experimental performance.

Results show that a 3C-SiC MOSFET designed for 600 V blocking exhibits a specific on-resistance of ~0.8 mΩ·cm^2^, corresponding to a drift-layer thickness of 7 μm and a doping of 1 × 10^16^ cm^−3^. This compares favorably with reported experimental data for 3C-SiC [21,33,38] and 4H-SiC [6,28,39,40,41,42,43], though 4H-SiC remains superior for ultra-high-voltage applications due to its higher breakdown strength.

**Table 5:** Comparison of specific on-resistance values: experimentally reported data from the literature and the results obtained in this work.

On-Resistance (mΩ-cm^2^)	Breakdown Voltage (V)	SiC Poly-Type	Reference
17–46	600	3C-SiC	[33]
5–7	600	3C-SiC	[5]
5–7	600	3C-SiC	[38]
0.392	360	3C-SiC	Device #l
0.84	535	3C-SiC	Device #2
2.42	990	3C-SiC	Device #3
1.8	660	4H-SiC	[43]
1.7	790	4H-SiC	[44]
5.1	1200	4H-SiC	[45]
5.59	1660	4H-SiC	[46]
5.5	1700	4H-SiC	[47]
14.2	3300	4H-SiC	[48]
13.5	3900	4H-SiC	[49]
6.95	1000	4H-SiC	[40]
5	1350	4H-SiC	[39]
20, 80	3300, 6500	4H-SiC	[50]

In these calculations, both contact and substrate resistances are assumed negligible. Reported ohmic contact resistances are typically ~10 μΩ·cm^2^ [28], far below the intrinsic MOSFET resistance. Similarly, with a thinned 2 μm n-type substrate (Nd = 10^19^ cm^−3^), substrate resistance is insignificant. For thicker substrates (e.g., 100 μm), resistance contributions can reach ~0.125 mΩ·cm^2^, but this is still much smaller than the MOSFET’s intrinsic resistance and two orders of magnitude below experimental device values.

The substrate resistance does not affect the specific on-resistance calculations considered in this study. Commercial SiC devices typically employ aggressive substrate thinning to minimize this resistance. For an n-type substrate doping concentration of 10^19^ cm^−3^, the bulk mobility is approximately 100 cm^2^/V.s. This corresponds to a negligible substrate resistance of about 12.5 μΩ for device No. 2 with a pitch size of 3.75 μm. Even at a blocking voltage of 1 kV, the substrate resistance is roughly four orders of magnitude smaller than the specific on-resistance of 2–3 mΩ·cm^2^ calculated for the MOSFETs. In all simulations, a 2 µm-thick substrate with an n-type doping of 1 × 10^19^ cm^−3^ is used across all pitch sizes. These calculations confirm that the substrate resistance is on the order of μΩ, and significantly smaller than the specific on-resistance values obtained.

## 4. Conclusions

In this study, comprehensive two-dimensional simulations were performed to investigate the behavior of 3C-SiC MOSFETs, focusing on the influence of channel mobility, drift region thickness, and doping concentration on breakdown voltage and specific on-resistance. Comparisons with simplified one-dimensional analytical models provided useful validation and offered a clearer picture of the physical mechanisms controlling device performance. Future work will incorporate calibration to experimentally measured IDS–VGS characteristics as consistent data matched to the relevant material quality, doping, and mobility conditions become available.

The results demonstrate that cubic silicon carbide (3C-SiC) exhibits distinct advantages for medium-voltage power devices. Its relatively higher channel mobility contributes to reduced on-state resistance at a blocking capability near 600 V, making it a promising candidate for applications such as power conversion systems, motor drives, and automotive electronics where low conduction losses are critical.

The analysis also underscores the importance of engineering the drift-layer profile—through optimized doping and scaling strategies—to achieve a favorable balance between resistance and breakdown strength. While this work is limited to room-temperature simulations, extending the study to incorporate temperature-dependent effects such as mobility degradation and ionization coefficients would provide a deeper understanding of the material’s performance under practical operating conditions.

A channel mobility sensitivity analysis represents a valuable optimization approach for device design as a potential direction to further enhance this work. Future work will incorporate calibration to experimentally measured on-state breakdown relationships as consistent data matched to the relevant material quality, doping, and mobility conditions become available.

Beyond the immediate case of vertical 3C-SiC MOSFETs, the insights gained here can be generalized to other wide-bandgap device structures, including lateral MOSFETs, JFETs, and even emerging designs such as trench-gate architectures. The same principles of mobility optimization, drift region engineering, and resistance–voltage trade-offs remain central across different geometries and material systems. As such, this work not only advances the understanding of 3C-SiC MOSFETs but also provides a design framework that can be applied to a broad range of power semiconductor technologies.

## Figures and Tables

**Table 1:** (**a**) Nominal material and device properties used in MOSFET simulations. (**b**) Material and physical constants.

(**a**)			
**Symbol**	**Parameter**	**Value (s)**	**Unit**
–	Source depth	0.25	µm
–	Source doping	1 × 10^19^	cm^−3^
–	Body depth	1	µm
–	Body doping (step profile)	1 × 10^18^ → 1 × 10^17^	cm^−3^
–	Pitch size	3.25, 3.75, 4.25	µm
–	Drift-layer thickness	4, 7, 12	µm
–	Drift-layer doping	2 × 10^16^, 1 × 10^16^, 0.5 × 10^16^	cm^−3^
–	Substrate doping	1 × 10^19^	cm^−3^
–	Substrate thickness	2	µm
t_OX_	Gate oxide thickness	0.05	µm
–	Channel doping	1 × 10^17^	cm^−3^
-	Channel depth	0.1	µm
L	Gate length	0.05	µm
W	Gate width	1	cm
(**b**)			
**Symbol**	**Parameter**	**Value**	**Unit**
χ	Electron affinity (3C-SiC)	4.05	eV
ΦM	Work function	4.0	V
Eg	Energy bandgap (3C-SiC)	2.4	eV
εr	Dielectric constant (3C-SiC)	9.66	–
εr(ox)	Dielectric constant (oxide)	3.9	–
q	Electronic charge	1.6 × 10^−19^	C
ε_0_	Permittivity of vacuum	8.854 × 10^−14^	F/cm

**Table 2:** Bulk mobility empirical model.

Symbol	Parameter	3C-SiC	4H-SiC	Unit
μ_max_	Maximum mobility	1100	900	cm^2^/V·s
μ_min_	Minimum mobility	0	20	cm^2^/V·s
α	Mobility fitting parameter	0.70	0.78	–
N_r_	Reference concentration	6 × 10^17^	2 × 10^17^	cm^−3^
E_br_ (at 10^16^)	Critical breakdown field	1.5	2.1	MV/cm
μ_channel_	Channel mobility (range)	100–370 *	20–180 *	cm^2^/V·s

* Largest experimental value reported.

**Table 3:** Impact ionization field parameters.

Equation (2)	Electrons	Holes
A [cm^−1^]	3.34 × 10^4^	4.46 × 10^6^, for *E* > 1.75 MV/cm 10.86 × 10^6^ for *E* < 1.75 MV/cm
B [MV/cm]	2.42	9.8, for *E* > 1.75 MV/cm 11.33 for *E* < 1.75 MV/cm

**Table 4:** Breakdown voltage calculations using 2D simulation for different pitch sizes.

Pitch size (μm)	3.25	3.75	4.25
Device Width (μm)	3077	2667	2353
Drift Layer doping (cm^−3^)	2 × 10^16^	1 × 10^16^	0.5 × 10^16^
2D-Drift Layer thickness (μm)	4	7	14
1D Model-depletion thickness (μm)	4.63	8.05	14.60
Breakdown voltage MOSFET (V)	360	535	990
R_on_ (mΩ-cm^2^)	0.392	0.84	2.42

## Data Availability

The original contributions presented in this study are included in the article. Further inquiries can be directed to the corresponding author.
